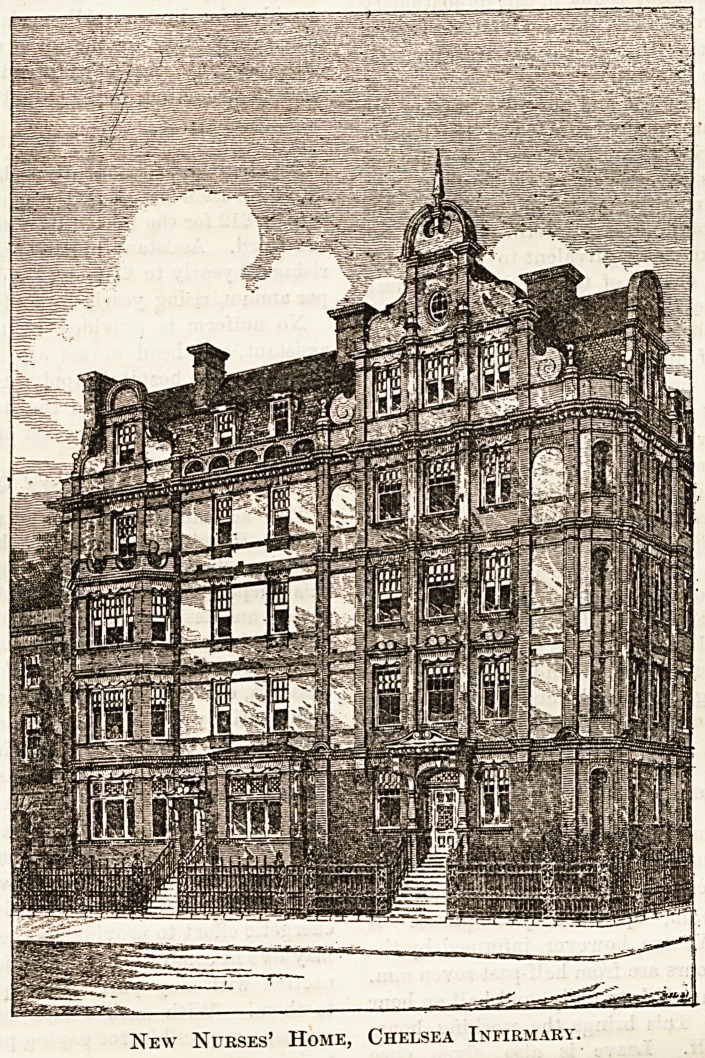# The Hospital Nursing Supplement

**Published:** 1896-11-07

**Authors:** 


"The Hospital" Nursing Mirror.

Being the Extra Nursing Supplement of "The Hospital."

[Contributions for this Supplement should be addressed to the Editor, The Hospital, 28 & 29, Southampton Street, Strand, London, W.C., and should have the word "Nursing" plainly written in left-hand top corner of the envelope.]

### News from the Nursing World.

### PRINCESS CHRISTIAN'S NURSES' HOME.

On Tuesday afternoon a dramatic performance was given at the Albert Institute, Windsor, under the special patronage of the Queen, in aid of Princess Christian's Nurses' Home at Windsor. H.R.H. Princess Christian was present herself, and with her were her two daughters, Princess Victoria of Schleswig-Holstein and Princess Aribert of Anhalt,

### THE DUCHESS OF TECK AT BRACKNELL.

Beautiful weather helped to make the Art Exhibition lately opened by Princess Mary Adelaide at the Victoria Hall, Bracknell, in aid of the funds of the Royal Berkshire Hospital at Reading, thoroughly successful. The Duke and Duchess of Teck were staying at Warfield Park with the Hon. Arthur and Lady Clementine Walsh, and were so pleased with their first visit to the exhibition that they paid another on the second day, thoroughly inspecting all the stalls and making a number of purchases.

### SELLY OAK DISTRICT NURSE SOCIETY.

The annual meeting of the District Nurse Society at Selly Oak, Birmingham, was held the other day, bad weather unfortunately preventing a large attendance. The report stated that 180 cases had been dealt with during the past year, the nurse paying 3,691 visits. The committee drew attention to the fact that the balance in hand on the year's working was a good deal less than last year, being £16 7s. 7d., whereas then it amounted to £28 0s. 6d.; they impressed upon subscribers the importance of inducing their friends to take an interest in the society. They reported in high terms of the work of the nurse, to whose "attention and perseverance" many good recoveries among serious cases were to be attributed.

### CLASSES FOR MASSEUSES.

Classes are being held at the Trained Nurses' Club, 12, Buckingham Street, Strand, in connection with the Society of Trained Masseuses, at which instruction will be given by Miss Dove in the "Physical Exercises and Movements Required in the Treatment of Curvature of the Spine." The training includes instruction in the general anatomy of the back, and demonstrations on the nature and different forms of spinal curvature. The classes are intended "to afford masseuses and intending masseuses the opportunity of qualifying to carry out under medical direction this most important physical treatment in spinal cases, combined with that of massage." Particulars can be obtained from Miss Dove, at the club.

### THE NEW INFIRMARY AT ISLEWORTH.

The new infirmary recently opened by the Duchess of Teck is an imposing building, and possesses a very nice nurses' home, though, as is too usual, space in this department has been cramped in a way which will certainly be found inconvenient before very long. Each nurse and probationer has a charming room to herself, of a good size, and as comfortably furnished as could be wished by the most critical. Each room has a fireplace, and is provided with a large cupboard from floor to ceiling, a combination dressing-table and chest of drawers, and a washstand. There is a cosy general sitting-room, and the dining-room, a very small apartment to be the only one for the use of a staff of twenty-two nurses, is in the central administration block. There are only two bathrooms in the home, one on each floor. A pleasant garden for the nurses' use will develop itself in course of time, where trees are being planted and order gradually introduced; at present everything is very new and not quite completed, for the infirmary has only been itself ready for occupation for two months or so. The matron has secured thoroughly trained women for her ward sisters, several of them coming from St. Marylebone Infirmary. Three of the staff are members of the Workhouse Infirmary Nursing Association, and these nurses were presented to the Duchess of Teck at her special request when she visited the infirmary at its opening.

### DISTRICT NURSING ON WHEELS.

It speaks much for the common sense of district nursing superintendents that the advantages of bicycles for district work have been so promptly and widely recognised, and sentimental objections have not been allowed to prevent their adoption. The saving of time and fatigue is enormous to the nurse, and more work can be got through, so the benefit is great to patients and nurses alike. Many committees of district nursing associations now provide machines as a matter of course for their nurses, examples which one may hope will be soon universally followed all over the country wherever wheeling is practicable.

### NURSING AT TAVISTOCK.

Boards of Guardians all over the country are finding it increasingly difficult to get satisfactory answers, or even answers at all, to their advertisements for nurses. At Tavistock a trained nurse has been advertised for to take charge of one side of the infirmary, but no applications resulted, and it was then decided to engage a nurse from the Workhouse Infirmary Nursing Associa- as soon as one could be supplied; but this wise proposal was subsequently objected to, and an amendment brought forward to advertise again, this time for an "assistant" instead of a certificated nurse. As the Board had already passed a resolution to engage a certificated nurse, this involved the rescinding of the former motion, and the matter remains in abeyance till another meeting. It can only be hoped that wiser counsel may in the end prevail, and a properly trained and competent nurse secured for the male wards, more especially as it would appear that the present nurse, who has so far been working single-handed, is "getting up in years."

### NEWCASTLE INFIRMARY NURSES' HOME.

The nursing staff at the Newcastle Infirmary have been suffering from insufficient accommodation for some time, and the committee have lately rented for them three houses in Ravensworth Terrace, Westgate Road, as temporarary quarters, pending the erection of the new infirmary and a proper home. The Mayor and Mayoress of Newcastle take much interest in the affairs of the infirmary, and the Mayoress has been especially exerting herself in raising money for the furnishing of these houses, which has cost between £400 and £500. The furniture has been bought with the view of future use in the new building. With the help of a committee of ladies half the amount has been secured, and a subscription ball at the Grand Assembly Rooms took place on November 3rd, which, it is hoped, will complete the sum wanted. Subscriptions towards the fund will be very gladly received by the Mayoress (Mrs. Albert Lord).

### NURSING IN CALIFORNIA.

We are constantly warning nurses of the difficulties into which they are likely to fall if they believe in and respond to representations of the good work and pay awaiting them in foreign lands. From the other side of the Atlantic come stories which go to prove that American nurses also fall victims to this desire for "pastures new." Trained nurses from Philadelphia and Baltimore have migrated to Southern California in the belief that they would there find employment and pay at the rate of twenty and twenty-five dollars a week, and have found to their cost that not only was there little or no work to be had, but what there was by no means commanded these abnormal fees. The San Diego Medical Society have published a statement of these facts, adding a resolution "for the information of such as may be hereafter likely to fall victims to such misrepresentation, that the profession of nursing is now greatly overstocked, and that at no time has any demand or such opportunities existed for trained nurses as represented."

### LINCOLNSHIRE NURSING ASSOCIATION.

On Monday last a very interesting gathering took place at Haverholme Priory on the occasion of the annual meeting of the committee and nurses of the Lincolnshire Nursing Association. The nurses, who are now working in all parts of the county, were fortunately all able to be present, with the exception of Nurse Caroline, who was detained by a serious case at Holbeach. The guests began to arrive about mid-day, and included the Master of St. Katharine's (President of the Queen Victoria Jubilee Institute for Nurses), to which the Association is affiliated; Miss Copeman, District Superintendent of Sister Katharine's Training Home at Plaistow, under whose supervision all nurses trained under County Council scholarships have been placed; Nurse Eleanor, from Miss Bromhead's Nursing Institution at Lincoln, by whom the candidates have, most of them, been personally examined as to their fitness before they are sent up for training, and some of the members of the committee of the Association. The nurses who were present consisted of seven Queen's Nurses and thirteen Rural Maternity Nurses, who have eceived six months' training under the County Council Scholarships, and very charming they looked in their neat, blue uniforms, each wearing the county badge, either of silver, or copper, according to their qualifications. After luncheon in the dining-room, the nurses dispersed about the house or grounds, whilst an informal meeting of the committee was held. After a short service in the chapel, followed by tea, Lady Winchilsea received each of the nurses separately in private to discuss with her the details of their work in their own districts. After dinner, at a quarter-past eight, Lord Winchilsea acknowledged the obligations under which the Lincolnshire Association lay to the Queen Victoria Jubilee Institute for inspecting its nurses, and thus assisting it to attain a high standard of efficiency. He expressed his sense of the value, both to the nurses and the association, of annually meeting together for friendly discussion, and hoped that they would always find it possible to do so once a year.

### GLOUCESTER DESIRES ANOTHER EPIDEMIC.

It is usual carefully to destroy wooden bedsteads and other material of the kind likely to retain the germs of any infectious disease, but the Gloucester sanitary authorities are so light-hearted—or light-headed—in their joy at the departure of the late epidemic of small-pox from their midst, that they are proposing now to distribute infected wood to all and sundry who will purchase it at a price, and the timber used in the construction of the late Isolation Hospital is, we hear, stacked up ready and waiting for auction. A policy so inhuman, so dangerous, and devoid even of the excuse attaching to money-making, for the sums to be realised by such a sale must be paltry and unimportant, is calculated to tempt Providence once again to attack this city with a heavy visitation of disease.

### COUNTESS OF WARWICK'S NEEDLEWORK GUILD.

The eighth annual distribution of clothes made by Lady Warwick's Essex Needlework Guild has been taking place at the Saffron Walden Town Hall. Some nine thousand garments have been sent in from the various branches, and these will be made up into parcels and dispatched to certain charities, Lady Warwick herself presiding over the selection.

### ST. VINCENT'S HOSPITAL, DUBLIN.

At the annual dinner of the medical staff and past students of St. Vincent's Hospital, Dr. M'Ardle, who replied to the toast of "St. Vincent's Hospital," took for his principal theme the progress of nursing. He believed that "the essence of charity had not shone forth in the hospital till now," because the nursing had never before reached anything like perfection. He considered its present position was due to the admirable nursing arrangements which now prevailed there. And then, going on to speak of the late meeting of the Irish Workhouse Association, he read a communication from "one of the most brilliant professional practitioners of Ireland," in which he spoke of fever patients being left to the mercy of pauper attendants at night, and urged that "pauper nursing should be absolutely abolished as a shame and disgrace." He would himself wish for the establishment of some central body which would see that properly qualified nurses were employed. Dr. M'Ardle's speech met with applause, and Dr. Grimshaw, President of the Irish Royal College of Physicians, who was present, pointed out that the Dublin Hospital Sunday Committee would not help hospitals where there was not proper nursing, and added his belief that great improvement had resulted, and that "the Dublin hospitals were as well nursed as any hospitals in the world."

### Hygiene: For Nurses.

By John Glaister, M.D., F.F.P.S.G., D.P.H.Camb., Professor of Forensic Medicine and Public Health, St. Mungo's College, Glasgow, &c.

### XXXI.—MICROBES—THEIR PLACE, POWER AND EFFECTS — FORMS OF MICROBES — THEIR NATURAL HISTORY—MODE OF PROPAGATION —NATURE OF INFECTIVE MATERIAL—CONTAGIUM—INFECTIVE MICROBES—THEIR INTIMATE ACTION ON THE HUMAN BODY.

The microscopic parasites which cause diseases of the infective and contagious type have been the victims of a shifting nomenclature. They have been called *microzoaria* (Gr.: *mikros*, small; *zoa*, animals) by De Blainville; *microphyta* (Gr.: *mikros*, small; *phyton*, a plant), *microzyma*, by Béchamp; *protista* (Gr.: *protistos*, the very first), by Haeckel, from which *protophyta* and *protozoa* are derivatives, and since 1878, *microbes* (Gr.: *mikros*, small; *bios*, life), by Sédillot; *bacteria*, by Cornil and Babés (Gr.: *baktron*, a stick); *schizomycetes* (Gr.: *schizo*, to cleave; *mykēs*, a fungus), by Wunsche; micro-organisms and germs. The name microbe has this advantage, that it does not commit the user to any theory regarding its genesis, or whether it belongs to the animal or vegetable kingdom. This name will be, therefore, adhered to by us.

Microbes are found everywhere in nature, in air, earth, water, food, and within and without our bodies. They operate in curious ways and in diverse places, conservatively and destructively, and are both the friend and foe of man. They rid the earth of dead and decaying matter which they themselves have brought into this condition; they produce profound chemical changes during these operations; in their grosser forms they produce alcoholic and acetous fermentations, make our sweet milk turn sour, our butter to become rancid, our bread to become mouldy; they produce food for the growth and sustenance of plants, and they cause various plant diseases; and they are the prime causes of all the so-called zymotic or infective and contagious diseases of man and the lower animals. Some knowledge of them is, therefore, of the greatest importance.

They are beyond the ken of ordinary vision; indeed, to study them aright, it is necessary to call to our aid the highest powers of the microscope. They require to be magnified from 800 to 1,500 times before we can understand how they grow, and what they are like. A fair average size of microbe measures 1-200,000th part of an inch, and it has been calculated that 400 millions of them could be comfortably accommodated side by side on one square-inch of surface. It is only by using the most delicate scientific measuring apparatus that they can be measured.

They are extremely fastidious regarding the kind of nutriment upon which they will grow and thrive. One will live healthily on one form of food which would starve another. Some demand albuminous material, others can only thrive when this substance is excluded. So, also, with other conditions. One, for instance, grows best at a low temperature, another, when the temperature is higher. Some can only grow where air is abundant (the *ærobic* kinds), others will only grow when air is excluded (the *anærobic* kinds). Their detection in the body or elsewhere is rendered easier by their susceptibility to dyeing or staining by aniline dyes; and even in this respect they differ, since one is susceptible to one colour of dye, and another to a different. From the foregoing facts, therefore, it will be apparent that their investigation demands laborious and painstaking research.

The forms of microbes also vary, upon which fact working classifications have been based. Such a classification is as follows, viz.: (1) The dot-like forms, called *micrococcus* (Gr.: *mikros*, small; *kokkos*, a cell or berry). They may appear as single dots (*cocci*), as double dots (*diplococci*), as four dots, like a wool-pack tied cross-ways with string, or as beads strung on a string (*streptococci*); (2) the rod-like form, called *bacillus* (Gr.: *baculum*, a stick or rod). Some of them, however, show sufficient departure from the rod-like form to entitle them to separate names; thus the bacillus of glanders is called *b. mallei*, from *malleus*—a hammer, being hammer-shaped; and that of cholera the *bacillus comma*, from its likeness to that mark of punctuation; and (3) the spiral, or corkscrew form, called *spirillum*. To the first form belong the microbes of fowl cholera, swine fever, measles, scarlet fever, small-pox, cow-pox, foot and mouth disease, pneumonia, erysipelas, puerperal fever, and others. To the second, those of anthrax, tubercle, glanders, enteric fever, cholera, diphtheria, tetanus or lock-jaw, leprosy, and others. And to the third, that of relapsing fever.

Mode of Propagation.—They propagate by throwing off seeds, or spores. In the dot-like form, the original cell simply divides, called *fission*. In the other forms, spores form in the interior of the mature microbe, and, roughly speaking, are shed as peas from a pod. The spores are even less in size than the parent microbes, and are more resistant to means employed for destroying them. For example, while the microbe is usually destroyed in boiling water, the spores may survive unless the boiling-process be kept up for a time. What, therefore, used to be called the microbe theory is now substantiated into a living fact which must be reckoned with at many points.

Infective Material.—It is by infective material that diseases are spread. It has been characterised by different names. From early ages, it was known that there existed a something by which disease was conveyed from one person or place to another. To this, various names have been given. It has been called *effluvium* (something which flowed forth), *miasma* (something which defiles), *influence* (as in influenza), *virus* (poison), *fomites* (fuel or touchwood), *infection* (Lat., *inficio*—to corrupt), and *contagium* (Lat., *con*—with; *tango*, I touch). These latter terms are used indiscriminately, and often synonymously. This is confusing, and wrong. Nomenclature in this requires to be made definite. Infective material, however, is essentially particulate in character, and may be borne by the air, or carried upon clothing or other media. So long as they are in contact with moisture, microbes are held in retention and cannot be liberated until the dampness is dispelled. Aerial diffusion is, therefore, only possible in the case of dried microbes or spores. Being particulate, infective matter cannot penetrate any interposing barrier, even of paper, and much less through walls and doors.

The length of time after infective material has left the body of an infected person during which it is capable of doing mischief is largely determined by its environment. Abundance of fresh air and sunlight quickly destroy it, absence of these tend to keep it active; hence microbes are most plentiful in the dust of darkest corners. Destruction of infective material, therefore, resolves itself into annihilation of microbes and their spores.

The microbes of the different infective diseases have elect seats of operative activity within and upon the body, from which, too, the infective matter is chiefly shed. Upon this basis they may roughly be divided into three classes, viz., (1) those in which the skin is chiefly involved; (2) those in which the upper internal mucous tract (the air passages, &c.) is involved; and (3) those in which the lower mucous tract is implicated (intestines, &c.). To the first class belong erysipelas, scarlet fever, measles, German measles, small-pox, chicken-pox; to the second, scarlet fever, measles, diphtheria, whooping-cough, mumps, ophthalmia, influenza, pneumonia, and tubercle of lung; and to the third, cholera, enteric fever, yellow fever, and puerperal fever. While the infective material in each infective disease may be found in all the discharges of the patient, it is most abundant at the elect seats of operation. It is shed from the body not only during the febrile, but also in the post-febrile, stages of the disease.

The evil effects which result from the action of microbes within the body are due to certain poisonous substances which are produced in the course of their growth and multiplication. They live upon the albuminous fluids of the body, and hence the new substances formed—being derivatives of albumen—are called *albumoses*, or because of their poisonous action, *toxines*. It is these toxines which, formed directly in the blood itself, or absorbed into the blood from the tissues, are the cause of the symptoms. For example, the bacillus of diphtheria is located solely in the throat, nasal, or air passages, and it is there that the toxines are formed; which toxines, however, on being absorbed into the blood of the patient, produce the profound physical depression, and, not infrequently, after-paralysis. The toxine of the tetanus microbe causes convulsive contractions of the muscles of the lower jaw. They are, therefore, as lethal in their action as strychnia or morphia.

### Poor Law Nurses and the Superannuation Act.

A journal called *The Councillor* last week amused itself and certainly entertained its readers by making a silly attack upon The Hospital because we did not print in full a long letter from Mr. Rutherglen, which, for some reason or other, he had chosen to send to us as well as to various other papers. We never opened our columns to all and sundry who chose to send letters on the subject of the Poor Law Superannuation Act, and certainly it is not our custom to print circulars. The editor of *The Councillor* must indeed be short of copy if he fancies that it is in accordance with journalistic methods for an editor to print everything that comes to hand. As for Mr. Rutherglen's letter, it contained much which we thought beside the mark; the rest we dealt with, as public news, in the way which seemed to us best.

The Night Superintendent and Deputy Matron of the Nottingham Borough Isolation Hospital writes: I have read Mr. Rutherglen's letter in the *Lancet* and the reply thereto which appears in The Hospital of the 24th inst. The objection to the Poor Law Superannuation Bill, so far as the interests of nurses are concerned, have been so ably set forth that there is apparently little more to be said. Nevertheless there are two points in Mr. Rutherglen's letter which I should like to notice, viz., that it is an insult to nurses to suppose them actuated by such mercenary motives that they would object to enter the Poor Law service under the present regulations; and further, that all who enter for the future, since they will do so with their eyes open, will have no cause for complaint. With regard to the first statement, although I repudiate on behalf of nurses generally, mercenary motives, yet they cannot, and ought not to be indifferent to their own interests. Nurses are a hard-working body of women, not too highly paid, and they may reasonably object on principle to an enforced contribution towards an object in the benefits of which they have hardly any chance of participating. That nurses are not as a class mercenary, but are ready and willing to contribute to help their less fortunate sisters is proved by the existence of the Benevolent Fund in connection with the National Pension Fund, which was commenced by donations and collections from nurses, and will, I hope next year be largely increased by the same means. I may here remark that the Pension Fund is admirably adapted to the needs of nurses, who are an ever-shifting community, and in my opinion no other organisation is required for them. With reference to my second quotation from Mr. Rutherglen's letter, I consider the argument a bad one. There are no conditions in any calling which might not be defended by saying that workers are to be found willing to submit to them. Occupations which destroy health are dangerous to life, or are so wretchedly paid as to make decent living an impossibility, yet find volunteers owing to the struggle for existence. And competition in the nursing world is keen enough to make nurses submit to the new regulations which, however, I sincerely hope may yet be amended so as to give them the liberty of choice in the matter. I do not of course for an instant wish to compare the lot of nurses with that of those unhappy "white slaves of England" who perforce sacrifice health and all that makes life worth living for a bare existence, but merely to illustrate my point that voluntary submission to certain conditions does not justify those conditions. I desire to do justice to Mr. Rutherglen's motives, which I am sure are good and generous, but I think he has not considered the matter from the standpoint of the nurses. As a nurse who trained in one of the largest Poor Law infirmaries, I take great interest in this question, and thoroughly endorse the views of the matron of the Marylebone Infirmary.

### Conference of Women Workers at Manchester.

The annual Conference of the National Union of Women Workers was held last week in Manchester, and one afternoon was devoted to the consideration of "the registration of midwives," two papers being read, one by Miss Rosalind Paget, hon. treasurer of the Midwives' Institute, the other by Sister Katherine, of Plaistow. Miss Paget's was an interesting paper, describing the present condition of affairs and reviewing the past history of midwifery and the efforts made to improve that condition. She dealt with the objections raised against registration, pointing out why midwives of some sort are a necessity, and showing that it was no question of midwives *versus* doctors, but midwives *versus* ignorant and untrained women. At present, no Act existing by which any standard of efficiency amongst midwives can be enforced, the lives of the most important part of the nation—its mothers—are still entrusted to persons who call themselves midwives, but who are in many cases absolutely untrained, the women of the working classes being powerless to find out whether the midwives they employ are adequately competent or dangerously ignorant.

Sister Katherine's paper upheld "The Employment of Midwives," because in many rural districts, where doctors are few and distances great, it is impossible for a medical man to attend every confinement; the poor cannot afford to pay under ordinary circumstances a fee which would adequately remunerate a medical man; that a midwife performs certain nursing and other duties which a doctor would not perform; that in ordinary cases a midwife can give all the assistance that is necessary; that in such cases there is a certain advantage in keeping midwifery separate from ordinary medical practice in consequence of the risk of contagion; and that some women prefer to be attended by women.

In the discussion which followed the reading of the papers, Miss Cropper, Mrs. Percy Boulnois, Mrs. Bedford Fenwick, and Dr. Annie McCall took part, the latter making an eloquent and convincing speech in favour of registration. The discussion showed the general feeling of the meeting to be strongly in favour of the registration of midwives, and the following resolution was passed unanimously: "That this meeting regards the absence of public provision for the education and supervision of midwives as productive of much fatal disease and serious suffering among the poor of this country, and urges upon Parliament the importance of passing some measure for the registration of midwives."

### Chelsea Infirmary; Opening of New Nurses' Home.

Wednesday in last week was a great day at the Chelsea Infirmary, when the new home which has been built for the nursing staff was formally opened by Mr. Douglas Hamilton-Gordon, Chairman of the Board of Guardians. A number of visitors gathered in the large recreation-room in the new ward block at half-past three, when the building was declared open by Mr. Gordon; a handsome silver-gilt key was presented to him by Mr. Lansdell, one of the architects. Mr. Gordon made a speech in which he dwelt on the duty of boards of guardians not only towards the aged and sick poor, but towards those who nursed them. He believed that there was no institution where the nursing work was more thoroughly and efficiently carried out than in the Chelsea Infirmary, thanks to the care of Dr. Moore, their medical superintendent, and of Miss de Pledge, their matron. He would say, in the presence of their excellent staff of nurses, that the comfort of those who care for the sick was due as a matter of right, and expediency, for it was not possible for them to do their work well without adequate comfort and regard to health. A few other speeches were made, in which complimentary and pleasant things were said of Miss de Pledge and her staff, and then the visitors dispersed to inspect the home.

New Nurses' Home, Chelsea Infirmary.

In the evening, Mr. and Mrs. Douglas-Gordon gave an enjoyable entertainment to the nursing staff and visitors in the home. The guests were received in the charming sitting-room on the ground floor, refreshments were served in the recreation and dining-room, and in the former recreation-room a stage was erected, and here the Cingalese stick dancers and jugglers went through some wonderful performances, watched with great interest by the audience; Miss Newman and Miss Gold sang, and Miss Mabel Hunt gave two skirt dances, which were much applauded.

The new home occupies the corner of Sydney and Cale Streets, and is connected on each floor with the newest block of the infirmary. It provides accommodation for fifty-five nurses, separate bedrooms for every member of the staff, most completely and comfortably furnished. The whole building is fireproof, and fitted throughout with electric light. There is on the ground floor a large dining and recreation-room, as well as a delightful sitting-room for the nurses. The latter is furnished in excellent taste, is a large, bright room, well supplied with easy chairs and sofas, a piano, and book-shelves. All the little bedrooms looked very dainty on the opening day with their ample and spotless new furniture, and prettily decorated by their occupants with flowers for the great occasion. The dressing-table chests of drawers have been specially made to fit the space in the window, all the furnishing being done by Messrs. Debenham and Freebody. The night-nurses' quarters are on the top floor, shut off by a sound-proof door, and Miss de Pledge's forethought for her nurses' comfort has contrived a special sitting-room on the next floor for the private use of the night staff, and for the day staff nurses in the evening, with a small kitchen or scullery close by where their meals can be prepared. This idea is a new one, and the quiet and convenience of it will be much appreciated by the Chelsea nurses. It will thus be possible for the night nurses to come down to their own sanctum without being obliged to put on uniform, and have their meal to themselves before going out, saving their time and adding much to their comfort. The new building also includes a self-contained house for the medical superintendent. Several members of the board have taken a keen personal interest in the details of the work as it proceeded, and the result certainly is one on which all concerned may be congratulated. The Chelsea nurses have now a beautiful home, fitted with every comfort. The architects are Messrs. Lansdell and Harrison of Islington. The total cost of the new building is rather more than £10,000.

### Death in Our Ranks.

Nurse Lydia Meadus died on Wednesday, October 28th, at the Nursing Institution, Northampton, of typhoid fever, contracted from a patient whilst in discharge of her duties. Nurse Meadus has been on the institution staff for more than seven years. Her death is a great grief to all her fellow workers, by whom she was held in great esteem.

### Nurses in 1896—Their Quarters, Hours, and Food.

[These articles exhibit the actual condition of affairs in the spring of the present year.]

### WEST LONDON HOSPITAL.

### I.—Terms of Training.

Probationers are received for training at the West London Hospital between the age of twenty-two and thirty, applications from candidates above the latter age being only entertained under exceptional circumstances. If approved by the matron, candidates are accepted for one month on trial, for which period they are required to prepay their board, and this month does not count in the probationary period. If appointed probationer three years' service is signed for, during which time board, lodging, washing, and salary are provided by the hospital.

Courses of lectures on medical and surgical nursing are given which probationers are required to attend. Certificates are not given for a less period than three years, and are not given until a nurse leaves the service of the hospital. At the end of the probationary three years, nurses are eligible for appointment as assistant nurses, a term which at the West London may be taken as equivalent to that of staff nurse, while the nurse in charge of the ward is called head nurse instead of sister, the more usual hospital designation. At the West London Hospital, where the staff is small, thirty being the usual number of nurses, assistant and head nurses are not always necessarily promoted from the probationers, but are appointed from outside candidates. Assistant nurses must be not less than twenty-four years of age, and are required to pass an examination as to health. They are eligible for the post of head nurse in the event of a vacancy. Head nurses must be not less than thirty years of age. The rules state that assistant and head nurses, as well as probationers, are required to refund to the committee of the hospital, "the proportion of wages paid them in respect of the annual holiday the year's service for which has not been completed, upon dismissal or leaving the service of the hospital for any cause whatever." Nurses and probationers are expected, besides making their beds and keeping their rooms tidy, to scrub them out once a fortnight. Ward work includes dusting.

### II.—Hours of Work and Times off Duty.

The printed rules state that the hours on duty are from half-past six to nine p.m. for all nurses alike, and the hours off duty therein printed are only "every other Sunday from half-past ten a.m. to half-past nine p.m.," qualified by the phrase "if circumstances permit." We are, however, informed by the matron that the working hours are from half-past seven a.m. to eight p.m., with one hour off duty daily, and half an hour each for dinner and tea. This brings the working hours down to ten and a half. Leave is also given once in the week from five to ten p.m. Nurses are expected to be in the hospital always by half-past nine p.m. on other occasions, except when special late leave is granted by the matron, and are not permitted to sleep away from the hospital on their days off. Night nurses are on duty from nine p.m. to half-past eight a.m., and are off duty after their half-past eight dinner in the morning until twelve, when they go to bed. A fortnight's holiday is the allowance to all the staff, probationers, assistant nurses, and head nurses. Friends are permitted to visit nurses at the hospital, under certain restrictions. Nurses are expected to write in a book kept for the purpose in the hall the times of their leaving and returning to the hospital when off duty. The rules are comprehensive as to the duties required of the nurses, one of those referring to the head nurse, providing that she "shall be on duty from half-past six o'clock a.m. to nine p.m., and shall be prepared for any emergency."

### III.—Meals.

Breakfast for the day nurses is at seven a.m., head nurses breakfasting at a quarter to eight. Dinners begin at twelve noon, tea is served at half-past four p.m., and supper at eight p.m. Night nurses breakfast at eight o'clock p.m., and dine at half-past eight a.m. Breakfast is a meat meal; hot meat, vegetables, and puddings are provided for dinner. All meals are served in the dining-room except those for the night nurses, who take each night with them into the ward bread and butter, with eggs or meat of some kind, for the night's requirements.

### IV.—Salaries and Uniform.

For their first trial month probationers are required to pay £2 2s. for their board, and when appointed are paid at the rate of £12 for the first year, £16 for the second, and £20 for the third. Assistant nurses' salaries begin at £24 a-year, rising £2 yearly to £30, and head nurses are paid at first £28 per annum, rising yearly by £2 to £36.

No uniform is provided by the hospital. Probationers, assistant, and head nurses are all alike required to pay for their own, the hospital supplying the materials. The washing for the staff is done in the hospital laundry, but nurses are expected to defray the cost of washing cuffs and bonnet strings.

### V.—Nurses' Quarters.

The accommodation for the nursing staff at the West London Hospital is probably as bad as any to be found in London. Three houses adjoining the hospital are used for this purpose, thrown together to form one. Here probationers and assistant nurses sleep two or three in small rooms, not divided even by screens or curtains, and unprovided with proper furniture. The nurses' dresses are hung in the passages, covered only with a curtain, the space at disposal being entirely inadequate. Head nurses have bed-rooms to themselves. There is only one dining-room for all the staff, and that is a gloomy apartment in the basement of one of the houses, while the sitting-room is equally dreary and inadequate. The head nurses have very small sitting-rooms off their wards. No hospital in London more urgently requires a proper home for its staff. New and splendid wards have been recently added to the hospital. It remains to make an energetic effort to provide for the nurses a home where they may live in comfort—a word which cannot be used in connection with any part of the quarters at present allotted to them. With such a lack of accommodation it is, of course, not possible for paying probationers to be taken for training.

### Presentation.

Miss Orchard, who has for five and a half years filled the position of Superintendent at Fusehill Workhouse Infirmary, Carlisle, and is now leaving to take up similar duties at St. Olave's Infirmary, London, was before her departure presented with a testimonial from some eighty guardians and other friends. The gift consisted of a handsome travelling clock and a purse containing £20, with a framed list of subscribers. Canon Waterton, who made the presentation, heartily wished Miss Orchard success in her new and larger sphere of work, and spoke of the improvements effected at Fusehill under her management.

### A Book and its Story.

### CLAUDE GARTON.*

"Claude Garton was very happy. His father, a simple country gentleman, after much deliberation, settled the perplexing question, 'What shall I do for my boy?' by deciding to make a doctor of him; and now Claude—with his father's benedictions and his mother's prayers still ringing in his ears—was being whirled swiftly in a racing express train towards the renowned and picturesque city of Dunburgh, at whose ancient University he was to enter upon the study of the science and art of medicine." The "Dunburgh" of this story is Edinburgh, and the volume before us describes the career of a medical student. The author makes no pretensions to literary style, but he is thoroughly acquainted with his subject, and has succeeded in producing what strikes us as a very vivid and realistic picture of the surroundings amongst which many of our most famous physicians have been educated. It is very easy to see the scenes in the infirmary, in the class-rooms, at the operating table, and in the dispensary are drawn from life; and we fancy that the professors in the Edinburgh School of Medicine are described under thinly-veiled pseudonyms. The students themselves are pictured in all their types—the hard-working youth who attends lectures and takes his degree of M.D.; the "wasters," who loaf about drinking bars and come to grief; and the "chronics," who year after year matriculate, but, from idleness or incapacity, never succeed in passing. Claude Garton is himself a hard-working student, and his career would have been uneventful but for certain incidents in his private life, which form the plot of the story. Before leaving England he had fallen in love with Edith Bennett, the daughter of a physician who practised in a country village near his home, and there had been an understanding between them that as soon as Claude was in a position to marry they should be engaged. But the young man is lured away by the attractions of a barmaid at a restaurant frequented by students. "Gipsy," the barmaid, "is a magnificent creature—tall, graceful, and refined looking"; and though Claude believes no woman can make him waver in his allegiance to Edith, yet he cannot resist the temptation of visiting the restaurant, and talking to this new divinity. About this time Edith's father dies, and she comes to Dunburgh as a nurse in the infirmary. Claude meets her at the house of a professor, and his affection, which had begun to cool, is almost quickened into the old glow of love. But the young nurse is busy at her work; he does not see much of her, and he hovers more and more about the bar over which "Gipsy" presides, until, at last, one Sunday, he makes an appointment with her; and then, carried away by his passion, he asks her point blank to marry him. Fortunately for Mr. Claude Garton he had met an honest woman. "When you know who and what I am," she replies, "you may wish your words unspoken." And Gipsy tells him everything. When a girl she had been engaged in a milliner's shop in Liverpool, where she had attracted the attention of an American whom she married. Her husband was supposed to be rich; but they had not been long in New York before she found that he was a notorious swindler, and, to make matters worse, already married. Having been deserted by this rogue, she had returned to England, and ultimately come to Dunburgh as a barmaid. Instead of being repelled by these disclosures, Garton becomes more infatuated than ever, and finally persuades Gipsy to promise that she will marry him as soon as he has taken his degree. His father, hearing of this entanglement, remonstrates, and for a time the young man sees nothing of his family. A reconciliation, however, is brought about, and Gipsy leaves her situation, and goes to live with an old lady who has already befriended her in Liverpool, and who warmly approves of the engagement. One day, when Garton's course of study is nearly completed, a patient enters the dispensary, where "Professor Baird" is in attendance, suffering from severe dislocation of the shoulder. It cannot be reduced without anæsthetics, and Garton is directed to administer chloroform. The operation is successful; but, while they were waiting for the patient to recover intelligence, Garton suddenly exclaims, "His breathing has stopped!" The man lay as if dead; but there was no loss of self-control on the part of the surgeon and his assistants.

"The foot of the table was tilted high, the head of the patient was drawn over the now lowered end, where it hung attached to its limp neck as if the latter were broken, whilst a ghastly and vacuous expression lent it a significantly cadaverous aspect. Claude held the under jaw in a peculiar manner, pressing the root of the tongue forward from below in such a way that the organ was forced out as far as it would go, and held the glottis as patent as could be." Artificial respiration was resorted to, and in the end animation was restored, and the patient, "all unconscious that for some time he had lain in the valley of the shadow of death, walked out into the street."

Soon after this Garton receives a telegram from Gipsy, who wishes to see him at once. He finds her in great distress, having seen the American swindler to whom she had been married. The man (Davis by name) had recognised her; she had managed to elude him, but she was sure that he was looking for her. Claude consoles and encourages her; but when he is about to leave her they look out of the window, and there is Davis watching the house from the opposite side of the street, and when Garton leaves he is followed by Davis, with whom he has an altercation and a struggle, in the course of which he recognises him as the patient who had so nearly died under chloroform at the dispensary. Davis is a strong man, but Garton suddenly remembers that his arm cannot yet have recovered. "He clutched his arm firmly, and, with a quick expert twist, shot the joint out of its socket." His enemy is at his mercy, and at this moment Gipsy rushes upon the scene. She had heard the sounds of the struggle, and had run after them. Davis greeted her as his wife. She asserts that he is married already, but he answers his first marriage was invalid, as his wife was a married woman at the time of the ceremony. A fierce dispute follows, which ends by Davis pulling out a revolver, and shooting Garton in the head, and running away. "And Claude Garton lay supine on the pavement, the snowflakes falling down upon his senseless face and an assassin's bullet in his brain."

This exciting incident is followed by a description, realistic in all details, of a dangerous operation by which the bullet is removed from the brain. Garton recovers, and the story of his convalescence, during which he is nursed by Edith Bennett, is again true to life. "How smoothly and silently every part of the complicated life of the great hospital went on, day after day, night after night, like some mighty beneficent machine, constructed and controlled by invisible powers, and working without jolt or jar."

But we must not exceed the space at our disposal, and shall leave the reader to find out for himself the manner in which the story is wound up, of which we shall only say that the interest is maintained to the last page, and that the end is somewhat startling. This volume is one which nurses and young medical men will read with interest and profit.

* "Claude Garton: A Study of Dunburgh University." By Thomas J. Henry, F.R.C.S.E. (Edinburgh: E. and S. Livingstone.)

### Everybody's Opinion.

### ORPHANS CAGED.

The Medical Officer to St. Mary's Home, Broadstairs, writes: The utterances of the Press have often surprised me, but I think I have never been so thoroughly astonished as by the remarkable editorial foot-note to my letter in The Hospital of October 31st. Now I do not wish to say anything unnecessarily harsh, and I am, therefore, willing to attribute the mutilation of the quotation from your "Medical Correspondent's" original letter to mere negligence, and not to disingenuousness, for I cannot believe it to have been intentional. But the omission, which is serious (for it involves the gist of the real issue between us), cannot be allowed. Your correspondent, in the sentence which immediately preceded your quotation, wrote that the cubicles at St. Mary's Orphanage "as a matter of fact bear a strong resemblance to the cages of a travelling menagerie." I have stigmatised this, and I stigmatise it again, is a wilful and dishonest misrepresentation. And as you have quoted from his letter, I will quote from my reply to it. "I say that he wilfully misrepresents what he saw, or what he said he saw, at St. Mary's Orphanage. When he has seen in travelling menageries wild beasts' cages which contain excellent and comfortable beds and bedding, together with necessary utensils and conveniences for the toilet, and when he has seen such cages decorated with cards, pictures, and ornaments, then, and not till then, will he be justified in saying that the cubicles at St. Mary's bear a 'strong resemblance,' or indeed any resemblance at all to 'the cages of a travelling menagerie.'" Surely, this was enough! Was it necessary to repeat what Lovelace has sung, that iron bars do not make a cage? Was there any need to hunt down the false analogy any further, and to point out that wild beasts' cages are wet and dirty; that they affront the nostrils, and that all they contain, besides their occupants, is some straw scattered over bare boards? Surely such details might have been left to the intelligence of your readers! These are the conditions which, had they existed, or had anything in the least degree like them existed, would have formed the "abominations" to which I referred —hypothetical "abominations" conjured up only by the distorted imagination of your correspondent. Had they been realities neither I nor any decent medical man would, as I said, have tolerated them for an hour. Apparently you have chosen to associate yourself with your "Medical Correspondent." Be it so. It is a great deal more your look out than mine. So far my contention has been with him. As your name is known I have had every wish to write respectfully as regards yourself; as to your anonymous friend this is not so necessary. But when you invite me to resign my appointment at St. Mary's on the strength of the vain imaginings and misrepresentations and mistakes of an anonymous correspondent, and on the production of a "rough" (very rough) sketch of some cubicles that I have never seen, it does seem to me that you are guilty of —— well, I will not take it too seriously. Let me regard it as a refinement of playful humour hardly to have been looked for in The Hospital, which, whatever its excellencies may be, cannot be called an amusing periodical. I am truly sorry to write in so contemptuous a vein. I grant it is not in the highest style of man. But with an opponent who, on point after point, so hopelessly gives himself away, it is really impossible to adopt any other method. And I must be permitted to say that I am not the assailant in this discussion, and that I have a right to defend myself with the weapons that come to hand most conveniently. Your correspondent has already been obliged to admit his mistake in one important matter. Let him go a step further and retract, and express his regret for the whole of the ill-considered attack he has made on the Sisters of the Church. He is, as he assures you, their friend—perhaps the kind of friend from whom one would prefer to be saved—so the small act of anonymous self-abnegation would not cost him very much.

*** We publish the above letter, but we do not care to argue with a correspondent who so easily lashes himself into so fine a fury. Mr. Raven said, "Do you suppose that the clergy of this parish would consent to minister to the inmates of St. Mary's Orphanage if abominations existed such as your medical correspondent asserts to exist?" We asked him to point out any error in the description, and this letter is the only response!—Ed. *T. H.*

### BREAY v. BROWNE.

Miss Henrietta C. Poole, Member of Council of R.B.N.A., Matron, East Lancashire Infirmary, Blackburn, writes: As you have given space for a lengthened defence of Sir J. Crichton-Browne's conduct as vice-chairman of the Royal British Nurse's Association, will you, of your courtesy, allow me, in as few words as possible, to show why Miss Breay claimed her right to propose her resolution at the last general meeting of that corporation, and, when refused, necessarily defended her right by legal means? For a long time past there has been a growing feeling of dissatisfaction among the older members of our Association, at what we consider a failure on the part of the Executive Committee to carry out the aims and objects for which our charter was granted, namely, "the mutual counsel, comfort, and support of persons practising as nurses." We have had personal experience that, on the contrary, these "persons" are the last who would be allowed by many of the gentlemen on the executive committee to "take mutual counsel," or to "support" one another. I can myself bear witness to having been silenced by Sir J. CrichtonBrowne, who used the name of Her Royal Highness, the President, for that purpose. In January, 1896, I went to London to attend a meeting of the council with the sole object of taking part in the discussion relative to the case of Miss Barlow, this being part of the business on the agenda for that day's meeting. The vice-chairman absolutely prevented me from speaking, though I claimed my right to do so. Under these circumstances, and being anxious to avoid the scandal of publicity, to which the committee objected in Miss Barlow's case, a number of matrons and nurses (sixty-nine in all) signed a resolution embodying their protest against (1) some of the glaring injustices done to members of the corporation; against (2) excessive expenditure; against (3) the violation of the charter and bye-laws in certain cases. Of those who signed, thirty-six were matrons, some of them holding important positions in London and the provinces. We made no attempt to get a large number of signatures, as we thought that the professional standing of many of those who signed would entitle them to a fair, if not a courteous, hearing. Miss Breay's name, Miss Beachcroft's, and my own will be found among those to whom the charter was originally granted. We could have induced others to sign whose names appear in the same list, but we forbore to ask them, wishing to avoid every appearance of party spirit. Miss Breay kindly acted as secretary, and was selected to present the protest at the general meeting. It is a matter of history that, notwithstanding the fact that the resolution, embodying this protest, appeared on the agenda, Sir J. Crichton-Browne, acting as chairman, refused to receive it, on what Mr. Commissioner Kerr has ruled were insufficient grounds. Miss Breay has had the courage to defend our rights in a court of justice, and I, for one, sincerely thank her for the course she has adopted, while regretting that such an unpleasant duty should have been forced upon her. In asking her to act for us we did not anticipate such a result, though, at the same time, we heartily endorse her action. Sir J. Crichton-Browne has spoken of us as "a small and discredited faction"; we may be a minority, but I think he has yet to learn how many we are, and I fail to see in what way we have "discredited" ourselves, except it be owing to the fact that our representative, Miss Breay, sought justice at the hands of an English jury, and obtained it at Sir J. Crichton-Browne's expense. "All things come to those who wait," and I believe our patience is not yet worn out, and that the time is rapidly coming when we may be allowed to manage our own affairs. It is all we ask for; but, now or then, we refuse to be deprived of our rights, and will claim them, if need be, by appeal to law. Perhaps it may be wise for me to state that, in thus writing, I am not acting as the mouthpiece of any person or persons, although I know I am expressing opinions which are held by a good many.

### Minor Appointments.

Hitchin Lying-in Charity.—Nurse M. N. Richardson has been appointed to the post of Midwife for the Hitchin Lying-in Charity. She received her training at the British Lying-in Hospital, Endell Street.

### Lockers as Larders.

It is quite wonderful that no reforming hand has yet been laid on a disgusting custom which still obtains at many hospitals, and is a standing disgrace to our much-vaunted progress in matters of hygiene and sanitation, a custom which surely no medical man or nurse can be found to defend, but which seems to die exceedingly hard even in the great London hospitals. The habit of patients keeping stores of bread, butter, tea, and sugar, and other oddments of food in the cupboards beside their bed, sometimes shared with the occupant of a neighbouring bed, and in close contact with clothes, brush and comb, slippers, soap and other toilet necessaries, is indefensible on every ground. Of course, this unwholesome and nasty plan exists at those hospitals where patients are still required to provide certain portions of their diet for themselves, but even where committees are not sufficiently enlightened to do away with this bad relic of old times, surely some other method of keeping the food brought in by the patients' friends might be resorted to. The mere idea of using butter which has been reposing in a ward cupboard, and become well impregnated with its atmosphere, for two or three days is revolting beyond words, and is felt to be so by the patients themselves. They must leave the hospital wards with a curious idea of what is there considered a desirable standard of cleanliness and nicety, truly!

"My bread and butter has a strong taste of that nasty smelling stuff they've just been dressing my arm with," said a patient at one of the best known London hospitals the other day, as she tried to enjoy her tea in spite of the flavour of iodoform left by the dressings which had been lying on the locker wherein lived her butter. And another woman, accustomed to fresh, sweet food in her country home, confessed herself to a visitor at another large general hospital in London as quite unable to fancy a meal of which the ingredients had to be kept beside her bed all day and night.

If members of committees at hospitals where this plan prevails would, when they visit the wards of their institution, open and look inside the patients' lockers, and put themselves in imagination for one moment in the place of those sick people, the end of this revolting and objectionable habit would surely soon follow, and the edict would go forth that it must cease at once and for ever. And medical men might, on their side, well help towards this reform by strong representations of the unwholesomeness of the present state of things as it exists in too many hospitals.

### Appointments.

### MATRONS.

West Highland Hospital, Oban.—Miss Ellen E. Martin has been appointed Lady Superintendent of this hospital. She received her training at the Royal Hospital, Edinburgh, where she remained on the staff for eight and a half years; after her training acting as head nurse of the ear, throat, and skin wards, night nurse of male surgical wards, and as extra head nurse.

Royal Eye Infirmary, Plymouth.—Miss Wetherell has been appointed Matron at this institution. She received some training at the London Hospital, and completed it at the South Devon and East Cornwall Hospital, Plymouth, remaining on the staff for eight and a half years; for the past five years in charge of the male medical ward. Her leaving is much regretted, and she carries with her to her new work many good wishes for success from her fellow nurses.

Queen's Hospital, Birmingham.—Miss Charlotte Elkington, who has just been appointed Lady Superintendent of the Queen's Hospital, Birmingham, has held since 1894 the important post of assistant matron at St. Thomas's Hospital. Miss Elkington was a Nightingale probationer at St. Thomas's, and for three years worked for the National and Metropolitan District Nursing Association, afterwards being appointed sister of Victoria Ward at St. Thomas's, and thence being promoted to the assistant matronship at that hospital.

### The Book World for Women and Nurses.

[We invite Correspondence, Criticism, Enquiries, and Notes on Books likely to interest Women and Nurses. Address, Editor, The Hospital (Nurses' Book World), 28 & 29, Southampton Street, Strand, London, W.C.]

### MAGAZINES OF THE MONTH.

The Autumn Number of Chapman's Magazine has an effective coloured exterior, but no illustrations. This periodical, as it describes itself, is a magazine of fiction, and its contents include a serial and three short stories—at least, only two of these are short stories, for Mr. Edward Ridley's tale runs well over 100 pages. "The Story of Aline" this is, of which, we learn, a fuller version is to be published shortly by Messrs. Chapman and Hall, with a sequel. Violet Hunt and Ernest Bramah are contributors also to this number.

A specimen copy of The Lady's Realm is before us for review. This is the first appearance of the new magazine, and all that has been said concerning the wonderful sixpennyworth is amply justified in the present volume; and we feel that its production has supplied a want long felt by the public. "'The Lady's Realm' will deal with all subjects of interest to ladies . . . it will touch on matters affecting Society . . . and will be essentially a magazine for the refined and cultured home." An index to the contents of the first number displays the names of many prominent writers of the day; there are short stories by S. R. Crockett, by W. E. Norris, Sarah Grand, and Rosa N. Carey, and a long and complete tale by Marie Corelli. "My Plaisaunce," written by the Countess of Warwick, and the Duchess of Somerset's "Consolations in a Garden," are other articles, which may well lay claim to placing "The Lady's Realm" among the leading journals of the day. Topical matters, too, find a full discussion, as several chapters are devoted to the great world and its fashions each month. The illustrations, as well as the letterpress, are of an unusual order of excellence, and Messrs. Hutchinson and Co., who are the publishers, are to be congratulated on the success of their new venture, the programme of the succeeding numbers of which are calculated even to surpass the present issue.

The English Illustrated Magazine for November appears in a dainty cover, which is not its least attractive feature. Here we find some excellent items, though gunpowder and Nelson are to the fore, as is also another subject of topical interest, "Dr. Nansen at Home," by Herbert Ward, which is profusely illustrated.

Number I. of Sunday Hours, a journal for boys and girls, published by the Religious Tract Society, has just been added to the ever-swelling list of illustrated weeklies. There is good reading in these pages, and the magazine contains a letter from Mr. Gladstone, testifying his hearty good wishes for a paper the special bent of which is towards promoting the observance of the Lord's Day.

The New Series of The Leisure Hour commences this month. Part I. is a decided improvement on the past numbers of a magazine which is always of an educative and instructive nature.

### Novelties for Nurses.

### A USEFUL WATCH FOR NURSES.

A reliable watch is an essential part of the equipment of every nurse, but those for which this quality is claimed are too often broken reeds, perpetually needing repair. Messrs. Joseph Heming, 28, Conduit Street, have sent to us on trial a delightful little silver watch especially suited for nurses, with lever escapement and seconds dial. We have had it in use for more than three weeks, and find it an excellent timekeeper. In silver or gun metal the price is £2 2s., including the engraving of initials on the back. In gold the price is £5 5s. The watch is a convenient size, looks neat and good, has the advantage of a very clear face, and appears to be just the watch that will be found serviceable and useful to nurses and to other people who do not want to go to great expense over this necessary of life, and yet require it to be very particularly dependable.

### For Reading to the Sick.

### THE ABIDING PRESENCE.

### Verses.

I NEED Thy presence every passing hour ;
What but Thy grace can foil the tempter's power?
Who like Thyself my guide and stay can be?
Through cloud and sunshine, Lord, abide with me.
I fear no foe with Thee at hand to bless ;
Ills have no weight, and tears no bitterness ;
Where is death's sting ? Where, grave, thy victory?
I triumph still, if Thou abide with me.

—*Hymns Ancient and Modern.*

My heart is chiming gladness o'er and o'er.
Sings on "God's everlasting love! what would'st thou more?"
Yes, one thing more ! to know it ours indeed,
To add the conscious joy of full possession !
O tender grace that stoops to every need !
This everlasting love hath found expression
In loving-kindness which hath gently drawn
The heart that else astray too willingly had gone.
We thirst for God, our heaven *is* above.
Earth has no gift our one desire to meet,
And that desire is pledge of His own love.

—*F. R. Havergal.*

Doth He answer, The Ancient of days?
Will He speak in the tongue and the fashion of men?
Nay, He spoke with them first; it was then
They lifted their eyes to His throne.
"They shall call on Me." "Thou art our Father, our God, Thou alone!"
For I made them, I led them in deserts and desolate ways,
I have found them a Ransom Divine;
I have loved them with love everlasting,—the children of men,—
I swear by Myself, they are Mine.

—*J. Ingelow.*

### Reading.

"Lo ! I am with you alway, even unto the end of the world." Such were the words of Jesus when He was just about to ascend to heaven. The mediatorial throne was in view—the harps of glory were sounding in His ears; but all His thoughts are on the pilgrim Church He is to leave behind. His last words and benediction are for *them.* "I go," He seems to say, "to heaven to my purchased crown—to the fellowship of angels—to the presence of my Father; but, nevertheless, 'Lo ! I am with *you* alway, even unto the end of the world.'"

How faithfully did the Apostles, to whom this promise was first addressed, experience its reality! Hear the testimony of the beloved disciple who had once leant on his divine Master's bosom—who "had heard, and seen, and looked upon Him." That glorified bosom was now hid from his sight; but does he speak of an absent Lord, and of His fellowship only as among the holy memoirs of the past? No! with rejoicing emphasis he can exclaim, "Truly our fellowship is with Jesus Christ."

Do dark providences and severe afflictions seem to belie the truth and reality of this gracious assurance? Are you ready to say, "If the Lord be indeed with us, why has all this befallen us?" Be assured He has some faithful end in view. By the removal of prized and cherished earthly props and refuges, He would unfold more of His own tenderness. Amid the wreck and ruin of earthly joys, which, it may be the grave has hidden from your sight, One nearer, dearer, and still would have you say of Himself, "The Lord liveth; and blessed be my Rock ; and let the God of my salvation be exalted."—From "*The Mind and Words of Jesus Christ.*"

### Where to Go.

QUEEN'S HALL, LANGHAM PLACE.—Mr. Bancroft will give a reading (as arranged by himself) of Charles Dickens's "Christmas Carol," in aid of the cancer wards of the Middlesex Hospital, on Monday, November 23rd, at eight o'clock. Tickets can be obtained from Mr. Melhardo at the hospital, or at the hall. Stalls, 5s.; grand circle, 2s. 6d.; balcony and area, 1s.

ROYAL BRITISH NURSES' ASSOCIATION.—The secretary requests us to state that the first demonstration on "Invalid Cookery" took place at 17, Old Cavendish Street, on Tuesday, November 3rd, at half-past two p.m., before a much interested audience. The course will be continued as follows: —Tuesday, November 10th, half-past two p m.: Boiled chicken, egg sauce, fried fillets of plaice, sweet omelet, boiled custard, macaroni and tomatos, lemonade. Tuesday, November 17th, at half-past two p.m.: Chicken broth, chicken panada, stewed eels, grilled chop, tapioca pudding, savoury omelet. Tuesday, November 24th, half-past two p.m.: Veal and sago broth quenelles, fried sole, wine jelly, scones, scrambled eggs, gruel. Tuesday, December 1st, half-past two p.m.: Meat jelly, stewed ox-tails, sole à la Maître d'Hôtel, milk jelly, porridge, poached eggs. The first sessional lecture will be delivered by Mr. Isenthal, on Thursday, November 26th, at eight p.m., the subject being "A Demonstration of the Present Stage of the Application of the Röntgen Rays to Surgical Diagnosis." Members are admitted free, the general public on payment of one shilling.

### Notes and Queries.

The contents of the Editor's Letter-box have now reached such unwieldy proportions that it has become necessary to establish a hard and fast rule regarding Answers to Correspondents. In future, all questions requiring replies will continue to be answered in this column without any fee. If an answer is required by letter, a fee of half-a-crown must be enclosed with the note containing the enquiry. We are always pleased to help our numerous correspondents to the fullest extent, and we can trust them to sympathise in the overwhelming amount of writing which makes the new rules a necessity. Every communication must be accompanied by the writer's name and address, otherwise it will receive no attention.

*Home Wanted.*

(40) Can you tell me of any institution to which a working man of 35 could be sent who is suffering from the after-effects of concussion of the brain? He has just been discharged from an accident hospital, and there is some hope of his ultimate recovery under careful treatment.—*Nurse M. C.*

Nurse Mabel Carter does not give any clue as to the way in which the patient she is inquiring about is suffering. Concussion of the brain leaves very different symptoms in different cases. If he is insane he should go to an asylum. If he is suffering from paralysis, or fits, it might be worth while sending him to a hospital—to the National Hospital for Diseases of the Nervous System, in Queen's Square. If only from debility, a convalescent home might be useful. A full list of such institutions will be found in Burdett's "Hospitals and Charities."

*Schott Treatment of Heart Disease.*

(41) Can you tell me where in London Schott's movements for the heart can be learned, and if there is any book on his method?—*Rice.*

Dr. Fletcher Little and Dr. Bezly Thorne are authorities on the Schott or Nauheim treatment, and there are pupils of the former who also give instruction. You might write to Miss Ellison, 258, Elgin Avenue, Maida Vale, on the subject. Are you a trained nurse? If not, we cannot advise you to study the treatment with a view to taking patients. Dr. Bezly Thorne is the author of a book on the treatment, "The Schott Method of the Treatment of Chronic Diseases of the Heart," published by Churchill.

*Advice Wanted.*

(42) A correspondent, "who has done a good deal of nursing, though she has never trained," asks our advice as to finding some employment which will enable her still to be with her husband, whose health is failing. She suggests taking a house at a higher rent in order to accommodate an invalid or elderly lady as boarder, and nurses for holidays or rest.

It is very difficult to advise, knowing so little of the circumstances, but it may be taken as a general rule that to enter upon an undertaking such as that suggested without the certainty of a good start is a very risky proceeding. A larger house means more furniture and increased payments, while boarders may not come, and advertising is expensive. To make such a venture on speculation cannot be advised. Would it not be possible to take in, perhaps, one boarder in the present small house, and thus avoid an initial outlay and its possible loss?

## Figures and Tables

**Figure f1:**